# Phenotypic profile and molecular mechanism of resistance in carbapenemase-producing Enterobacterales and *Pseudomonas aeruginosa* isolates from Brazilian hospitals: implications for the introduction of imipenem-relebactam

**DOI:** 10.3389/fmicb.2025.1689777

**Published:** 2025-11-14

**Authors:** Pedro Kurtz, Pedro F. Del Peloso, Bruno Rocha Pribul, Arthur M. Albuquerque, Bianca B. P. Antunes, Grazielle Vianna Ramos, Fernando A. Bozza

**Affiliations:** 1 Dor Institute of Research and Education, Rio de Janeiro, Brazil; 2 Intensive Care Department, Instituto Estadual do Cérebro (IECPN), Rio de Janeiro, Brazil; 3 Richet Laboratory, Rio de Janeiro, Brazil; 4 Instituto Oswaldo Cruz (IOC), FIOCRUZ, Rio de Janeiro, Brazil; 5 School of Medicine, Universidade Federal do Rio de Janeiro, Rio de Janeiro, Brazil; 6 Department of Industrial Engineering (DEI), Pontifical Catholic University of Rio de Janeiro (PUC-Rio), Rio de Janeiro, Brazil; 7 Instituto Nacional de Infectologia Evandro Chagas (INI), FIOCRUZ, Rio de Janeiro, Brazil; 8 Comprehensive Health Research Center (CHRC), NOVA Medical School, Universidade Nova de Lisboa, Lisboa, Portugal

**Keywords:** multi-drug resistant, imipenem-relebactam, gram-negative, antimicrobial therapy, carbapenemase

## Abstract

**Background/Objectives:**

Carbapenemase-producing Enterobacterales and *P. aeruginosa* are critical threats to global public health, especially in high-burden regions such as Brazil. Imipenem-relebactam (IMR), a combination of a carbapenem with a β-lactamase inhibitor, is a promising treatment option against resistant Gram-negative bacteria. This study aimed to characterize phenotypic resistance and molecular mechanisms in clinical isolates from Brazilian hospitals and assess IMR activity.

**Methods:**

A prospective multicenter study was conducted across 12 hospitals in Rio de Janeiro. A total of 150 Enterobacterale*s* and 100 *P. aeruginosa* isolates resistant to carbapenems were collected. Isolates were identified by MALDI-TOF and screened for carbapenemase genes (KPC, NDM, VIM, IMP, OXA-48) using PCR. Susceptibility to IMR was determined by broth microdilution following EUCAST guidelines. Next-generation sequencing (NGS) was performed on a subset of multidrug-resistant isolates.

**Results:**

IMR resistance was identified in 34.5% of *K. pneumoniae* and 74% of *P. aeruginosa* isolates. Among Enterobacterales, 21.1% of KPC-producers and 88.9% of OXA-48-producers were resistant to IMR. The bla_KPC gene was predominant, but NDM was increasingly detected. In *P. aeruginosa*, resistance was largely unrelated to carbapenemase production, implicating porin loss and efflux pumps. NGS revealed extensive co-resistance and multiple virulence genes in *K. pneumoniae* isolates.

**Conclusion:**

This study highlights the emergence of significant resistance to imipenem-relebactam in Brazil, driven by both enzymatic and non-enzymatic mechanisms. Ongoing molecular surveillance and tailored treatment strategies are essential to address the evolving threat of multidrug-resistant Gram-negative infections in endemic regions.

## Introduction

The global spread of antibiotic-resistant bacteria represents one of the most significant threats to public health today. Among Gram-negative pathogens, Enterobacterales and *Pseudomonas aeruginosa* (*P. aeruginosa*) producing carbapenemases stand out for their elevated resistance to carbapenems, a class of antibiotics frequently used as a last resort for severe infections. In Brazil, this situation is particularly concerning, with widespread reports of resistant isolates across multiple healthcare institutions (Kiffer et al., 2023).

Carbapenemases are enzymes capable of hydrolyzing carbapenems, rendering these antibiotics ineffective. Clinically relevant carbapenemases are classified into three main classes: class A (serine carbapenemases, including *K. pneumoniae* carbapenemase, KPC), class B (metallo-β-lactamases, such as NDM), and class D (OXA-48-like β-lactamases) (Tzouvelekis et al., 2012). The dissemination of these enzymes, particularly among *Klebsiella pneumoniae (K. pneumoniae)* isolates, has complicated treatment strategies, as these bacteria often display resistance to multiple antibiotic classes, including aminoglycosides, polymyxins, and tigecycline (Monteiro et al., 2009).

The development of new therapeutic agents is critical to addressing this challenge. Imipenem-relebactam (IMR), a combination of a carbapenem with a β-lactamase inhibitor, has emerged as a promising therapeutic option. Relebactam has the capability to restore imipenem activity against isolates that produce certain β-lactamases, offering renewed hope for the treatment of infections caused by these multidrug-resistant pathogens. Imipenem-relebactam pairs imipenem/cilastatin with relebactam, a diazabicyclooctane β-lactamase inhibitor that inhibits Ambler class A (including KPC) and class C (AmpC) enzymes but not class B metallo-β-lactamases (MBLs) or class D OXA-48-like enzymes. Consequently, imipenem-relebactam restores imipenem activity against many KPC-producing Enterobacterales and against some non-carbapenemase mechanisms (e.g., porin alterations with AmpC derepression), but it is ineffective against MBL- and OXA-48-like producers. Clinical data and contemporary in-vitro surveillance support IMR’s role in infections due to KPC-predominant CRE and difficult-to-treat *P. aeruginosa* (Motsch et al., 2020; Titov et al., 2021). However, few studies have systematically evaluated the efficacy of IMR against a broad range of clinical isolates, especially in regions with high resistance prevalence, such as Brazil (Hackel et al., 2018).

The COVID-19 pandemic may have influenced the epidemiological dynamics of carbapenem resistance in Brazil. This may have been the result of increased consumption of broad-spectrum antibiotics in patients with nosocomial bacterial complications after COVID-19 infection and more permissive antimicrobial stewardship practices (Antunes et al., 2023, 2025). Between 2015 and 2022, a marked shift in the prevalence of resistance genes among Enterobacterales was observed, with the blaKPC gene showing a reduction from 74.5% in 2015 to 55.1% in 2022. Conversely, the blaNDM gene exhibited a significant increase, rising from 4.1% in 2015 to 39.4% in 2022. This rise is particularly concerning given the broad-spectrum resistance conferred by NDM, which includes resistance to novel β-lactam/β-lactamase inhibitor combinations (Carvalho-Assef et al., 2013; Kiffer et al., 2023). Notably, *K. pneumoniae*, a member of the high-priority ESKAPE group, is considered highly prevalent among Enterobacterales.

In *P. aeruginosa*, carbapenem resistance remains a major clinical concern, prompting its inclusion in the World Health Organization’s list of critical-priority pathogens for the development of new antibiotics (World Health Organization, 2017). This pathogen possesses a wide array of resistance mechanisms, including β-lactamase production, porin modifications, and efflux pump overexpression, all of which contribute to its resilience in clinical settings (Poole, 2011). Over the last decade, several BL/BLI combinations have expanded options against multidrug-resistant Gram-negative bacteria. Ceftazidime–avibactam (CZA) is active against KPC- and many OXA-48-like-producing Enterobacterales, while meropenem–vaborbactam (MEV) is optimized for KPC-producing Enterobacterales. Ceftolozane–tazobactam (C/T) provides potent antipseudomonal activity, including against difficult-to-treat *P. aeruginosa*, though it lacks activity against carbapenemase producers. Current guidance emphasizes mechanism-directed selection of these agents based on local epidemiology and rapid genotypic/phenotypic testing (Tamma et al., 2024; Shortridge et al., 2023).

This study aims to investigate and characterize the mechanisms of carbapenem resistance in isolates of Enterobacterales and *P. aeruginosa* from Brazilian hospitals, focusing on the efficacy of IMR as a potential therapeutic option against these resistant pathogens. Specific objectives include: (1) Quantify the prevalence of clinically relevant isolates of Enterobacterales and *P. aeruginosa* resistant to carbapenems in various Brazilian hospitals; (2) Identify and describe the primary molecular mechanisms of resistance present in these isolates, including the detection of carbapenemase genes (e.g., KPC, NDM, VIM, IMP, OXA-48) using PCR and next-generation sequencing (NGS); (3) Investigate the presence of new or rare resistance mechanisms in isolates that lack common carbapenemase genes through genomic sequencing and bioinformatics analysis.

## Methods

### Study design

This study was conducted as a prospective, multicenter study involving the collection and analysis of clinical isolates of Enterobacterales and *P. aeruginosa* from 12 hospitals in Rio de Janeiro, Brazil. The study was carried out over a 1-year period (1 January 2021, through 31 December 2021), encompassing initial collection, molecular, and microbiological analyses.

Clinical isolates of Enterobacterales and *P. aeruginosa* were obtained from hospitalized patients demonstrating carbapenem resistance. Only one isolate per patient was considered to avoid duplication of data. Isolates were collected from blood, respiratory secretions, urine and other sources. Isolates were identified at the genus and species level using the VITEK® MS MALDI-TOF system (bioMérieux, France), a technology based on matrix-assisted laser desorption ionization time-of-flight mass spectrometry (MALDI-TOF). We screened for a broad panel of carbapenemase genes, including KPC, NDM, OXA-48-like, and the metallo-β-lactamases VIM, IMP, and SPM-1, using polymerase chain reaction (PCR) with specific primers and standardized amplification protocols. The susceptibility profile for imipenem–relebactam was determined by reference broth microdilution using the Sensititre™ Gram Negative RUO Susceptibility Testing Plate - MDRGNXXF (Thermo Fisher Scientific), interpreted by The European Committee on Antimicrobial Susceptibility Testing (2024) breakpoints. Quality control used appropriate reference strains and CLSI/EUCAST procedures (The European Committee on Antimicrobial Susceptibility Testing, 2024). Isolates that were negative for common carbapenemase genes but still resistant to carbapenems underwent next-generation sequencing (NGS) for the identification of novel resistance genes or mutations. Isolates resistant to carbapenems, polymyxin B, and fluoroquinolones were selected for NGS to identify resistance genes, virulence factors, and genetic structures. Libraries were prepared with Nextera XT and sequenced on an Illumina MiSeq (2 × 250 bp). Reads were trimmed (Trimmomatic) and assembled (SPAdes v3.14). Assemblies were screened for resistance genes (ResFinder 4.1), plasmid replicons (PlasmidFinder), and MLST. Kleborate assessed hypervirulence loci for *K. pneumoniae; in P. aeruginosa* we examined oprD and efflux regulators (e.g., mexR, nalD).

### Statistical analysis

We focused on descriptive analyses. We used the median and interquartile range (IQR) for continuous variables and frequency and proportions for categorical variables. Prevalence data, resistance profiles, and MIC results were analyzed using appropriate statistical methods. Associations between resistance genes and IMR efficacy were assessed using chi-square tests and logistic regression. The analyses were performed using R 4.3.2 (R Core Team 2023).

## Results

Most isolates were recovered from respiratory tract specimens (e.g., ventilator-associated pneumonia), accounting for 58% of isolates, followed by bloodstream isolates (32%), and a smaller fraction from urine or other sources (10%). Among the 150 Enterobacterales and 100 *P. aeruginosa* clinical isolates analyzed, high rates of phenotypic resistance to carbapenems were observed. In *K. pneumoniae*, 69% and 76.1% of isolates were resistant to imipenem and meropenem, respectively. Resistance to IMR was detected in 34.5% of isolates. For *P. aeruginosa*, resistance to imipenem and meropenem reached 87% and 67%, respectively, with 74% demonstrating resistance to IMR. Notably, *S. marcescens* exhibited the highest imipenem resistance rate among Enterobacterales (90.9%), while *E. coli* showed a much lower rate (12.5%) (Table 1).

**Table 1:** Phenotypic resistance profile of Enterobacterales and Pseudomonas species.

Bacterial species	*N*	Resistance (*N*, %)				
		Ceftazidime	Colistin	Imipenem	Meropenem	IMR
*Klebsiella pneumoniae*	113	40 (35.4%)	38 (33.6%)	78 (69%)	86 (76.1%)	39 (34.5%)
*Pseudomonas aeruginosa*	100	42 (42%)	68 (68%)	87 (87%)	67 (67%)	74 (74%)
*Serratia marcescens*	11	5 (45.5%)	9 (81.8%)	10 (90.9%)	11 (100%)	8 (72.7%)
*Escherichia coli*	8	3 (37.5%)	2 (25%)	1 (12.5%)	2 (25%)	3 (37.5%)
*Klebsiella oxytoca*	6	3 (50%)	3 (50%)	3 (50%)	3 (50%)	5 (83.3%)
*Morganella morganii*	2	1 (50%)	2 (100%)	1 (50%)	0 (0%)	2 (100%)
*Citrobacter freundii*	2	1 (50%)	0 (0%)	1 (50%)	1 (50%)	1 (50%)
*Providencia stuartii*	1	1 (100%)	1 (100%)	1 (100%)	1 (100%)	1 (100%)
*Proteus mirabilis*	1	1 (100%)	1 (100%)	0 (0%)	0 (0%)	0 (0%)
*Enterobacter cloacae*	7	2 (28.6%)	1 (14.3%)	4 (57.1%)	3 (42.9%)	5 (71.4%)
*Klebsiella aerogenes*	1	1 (100%)	0 (0%)	1 (100%)	1 (100%)	1 (100%)

IMR, imipenem-relebactam.

Molecular analysis revealed the presence of several carbapenemase genes. In *K. pneumoniae*, the *blaKPC* gene was detected in 60 isolates, *blaNDM* in 18, and *blaOXA-48* in 6. Among *P. aeruginosa*, only three isolates carried *blaKPC* and one had *blaNDM*. The overall predominance of *blaKPC* among Enterobacterales reflects its role as the main resistance mechanism in this setting. No isolates tested positive for *blaIMP* or *blaVIM*. No isolate was found to carry the *blaSPM* (SPM-1) metallo-β-lactamase gene (Table 2). Notably, no isolate was found to carry more than one carbapenemase gene simultaneously (e.g., we did not observe co-production of KPC and NDM in the same strain).

**Table 2:** Molecular mechanisms of resistance of carbapenemases.

Bacterial Species	*N*	KPC Gene	NDM Gene	OXA-48 Gene	IMP Gene	VIM Gene
*Klebsiella pneumoniae*	113	60	18	6	0	0
*Pseudomonas aeruginosa*	100	3	1	0	0	0
*Serratia marcescens*	11	5	1	0	0	0
*Escherichia Coli*	8	2	2	0	0	0
*Klebsiella oxytoca*	6	2	1	0	0	0
*Morganella morganii*	2	0	1	0	0	0
*Citrobacter freundii*	2	1	2	0	0	0
*Providencia stuartii*	1	1	0	1	0	0
*Proteus mirabilis*	1	0	0	0	0	0
*Enterobacter cloacae*	1	5	1	2	0	0
*Klebsiela aerogenes*	1	0	0	0	0	0

Analysis of resistance profiles specifically to IMR revealed that among KPC-producing Enterobacterales, 21.1% were resistant. In contrast, 88.9% of OXA-48 producers were resistant to IMR. All three KPC-producing *P. aeruginosa* isolates tested were susceptible to IMR (Table 3).

**Table 3:** Resistance profile to imipenem-relebactam of carbapenemase-producing Enterobacterales and Pseudomonas.

Bacteria	Gene	Gene (+)	IMR *S*	IMR *R*	% *R*
Enterobacterales	KPC	76	60	16	21.1%
Enterobacterales	OXA-48	9	1	8	88.9%
*Pseudomonas aeruginosa*	KPC	3	3	0	0.0%
*Pseudomonas aeruginosa*	OXA-48	0	0	0	—

IMR, imipenem-relebactam; *S*, sensitive; *R*, resistant.

Next,-generation sequencing (NGS) performed on a subset of multidrug-resistant *K. pneumoniae* and *K. oxytoca* isolates revealed the presence of multiple resistance determinants. We selected 20 isolates (15 carbapenemase-producers spanning KPC, NDM, OXA-48-like, VIM, IMP; and 5 carbapenemase-negative) for WGS. All sequenced *K. pneumoniae* isolates carried *blaKPC-2* or *blaKPC-3*, and co-harbored ESBL genes such as *blaSHV-11* and *blaCTX-M-14*. Resistance to aminoglycosides [*aac(6′)-Ib3*], colistin (*pmrB_R256G*), and quinolones (*gyrA_S83I, parC_S80I*) was also common (Table 4).

**Table 4:** Analysis of next-generation sequencing resistance genes.

Species	ESBL genes	Beta-lactam resistance	Carbapenem resistance	Aminoglycoside resistance	Colistin resistance	Quinolone resistance	Other resist genes
*Klebsiella pneumoniae*	*blaSHV-11, blaCTX-M-14*	*blaTEM-1*	*blaKPC-2*	*aac(6′)-Ib3*	*pmrB_R256G*	*gyrA_S83I, parC_S80I*	*sul1, sul2, mph(A), fosA*
*Klebsiella pneumoniae*	*blaSHV-11, blaCTX-M-14*	*blaTEM-1*	*blaKPC-2*	*aac(6′)-Ib3*	*pmrB_R256G*	*gyrA_S83I, parC_S80I*	*sul1, sul2, mph(A), fosA*
*Klebsiella pneumoniae*	*blaSHV-11, blaCTX-M-14*	*blaTEM-1*	*blaKPC-2*	*aac(6′)-Ib3*	*pmrB_R256G*	*gyrA_S83I, parC_S80I*	*sul1, sul2, mph(A), fosA*
*Klebsiella pneumoniae*	*blaSHV-11, blaCTX-M-14*	*blaTEM-1*	*blaKPC-2*	*aac(6′)-Ib3*	*pmrB_R256G*	*gyrA_S83I, parC_S80I*	*sul1, sul2, mph(A), fosA*
*Klebsiella pneumoniae*	*blaSHV-11, blaCTX-M-14*	*blaTEM-1*	*blaKPC-3*	*aac(6′)-Ib3*	*pmrB_R256G*	*gyrA_S83I, parC_S80I*	*sul1, sul2, mph(A), fosA*
*Klebsiella pneumoniae*	*blaSHV-11, blaCTX-M-14*	*blaTEM-1*	*blaKPC-2*	*aac(6′)-Ib3*	*pmrB_R256G*	*gyrA_S83I, parC_S80I*	*sul1, sul2, mph(A), fosA*
*Klebsiella oxytoca*	—	*blaOXY-5-2*	*blaKPC-2*	*aac(6′)-Ib3*	—	—	*fosA, oqxB*

Virulence gene analysis identified several important markers in *K. pneumoniae*, including *ybtP* and *ybtQ* (iron acquisition), *magA* and *rmpA* (capsular synthesis and immune evasion), and *fimH* and *mrkD* (adhesion). Some isolates also carried *traT*, a serum resistance gene, and *irp2*, a siderophore biosynthesis component. *K. oxytoca* presented a similar profile, with *iutA* and *rmpA* among the detected virulence factors (Table 5).

**Table 5:** Analysis of next-generation sequencing virulence genes.

Species	Iron acquisition	Capsule and immune evasion	Adhesion and biofilm formation
*Klebsiella pneumoniae*	*ybtP*, *ybtQ* (Yersiniabactin—Iron acquisition system)	*magA* (Capsule polysaccharide synthesis), *rmpA* (Regulator of mucoid phenotype)	*fimH* (Type 1 fimbriae adhesion), *mrkD* (Type 3 fimbriae adhesion)
*Klebsiella pneumoniae*	*ybtP*, *ybtQ*	*rmpA*, *magA*	*fimH*, *mrkD*
*Klebsiella pneumoniae*	*ybtP*, *ybtQ*	*rmpA*, *magA*	*fimH*, *mrkD*, *traT* (Serum resistance)
*Klebsiella pneumoniae*	*ybtP*, *ybtQ*, *irp2* (Siderophore biosynthesis)a	*rmpA*, *magA*	*fimH*, *mrkD*, *traT*
*Klebsiella pneumoniae*	*ybtP*, *ybtQ*, *irp2*	*rmpA*, *magA*	*fimH*, *mrkD*, *traT*
*Klebsiella pneumoniae*	*ybtP*, *ybtQ*, *irp2*	*rmpA*, *magA*, *kfu* (Iron uptake system)	*fimH*, *mrkD*, *traT*
*Klebsiella oxytoca*	*iutA* (Aerobactin receptor)	*rmpA* (Mucoid phenotype regulator)	*fimH*, *mrkD*

## Discussion

Our findings provide a comprehensive overview of the phenotypic and molecular profiles of resistance to IMR among Enterobacterales and *P. aeruginosa* isolated from Brazilian hospitals. Compared to previous national data, this study highlights an alarming persistence of high resistance rates, particularly in *K. pneumoniae* and *P. aeruginosa*, two species commonly associated with healthcare-associated infections.

The resistance rates observed in this study align partially with earlier reports, where the prevalence of *bla_KPC* remained high but declining, and *bla_NDM* showed a growing trend. In our analysis, bla_KPC was detected in over 50% of *K. pneumoniae* isolates, confirming its status as the dominant carbapenemase in Brazil. However, the rising presence of *bla_NDM* is concerning due to its broad resistance spectrum and the inability of relebactam to inhibit metallo-β-lactamases (MBLs). This mirrors global trends: regions with increasing incidence of NDM and other MBLs (notably parts of Asia) have seen IMR efficacy markedly reduced, whereas areas dominated by KPC-type carbapenemases still report high susceptibility rates to this combination (Tängdén and Giske, 2015). Our findings place Brazil in this evolving context – historically a KPC-endemic setting now encountering a surge of MBL producers – underscoring that the utility of IMR may decline as MBL prevalence rises.

Phenotypically, resistance to IMR was observed in approximately one-third of *K. pneumoniae* and nearly three-quarters of *P. aeruginosa* isolates. These figures are notably higher than those reported in global surveillance studies. For example, the SMART surveillance program reported >96% of Enterobacterales isolates from Latin America to be susceptible to IMR, and the RESTORE-IMI 1 trial also found IMR retained significant activity against KPC-producing organisms (Haidar et al., 2017; Motsch et al., 2020; Carvalhaes et al., 2020; Livermore et al., 2020). The discrepancy in our study likely reflects the unique resistance pressures in Brazilian hospitals and the enriched nature of our sample (focused on carbapenem-resistant isolates). It may also relate to post-pandemic antibiotic utilization patterns, where increased broad-spectrum antibiotic use could have accelerated selection for IMR-resistant strains (Kanj et al., 2022; Tängdén and Giske, 2015). Overall, while IMR remains highly active against Gram-negative pathogens in many global contexts, our data reveal a substantial resistant subset emerging in Brazil, highlighting the importance of localized surveillance. Importantly, the gap between our resistance rates and those in broader surveillance underscores the contribution of specific mechanisms – particularly MBLs and OXA-type carbapenemases – that are present at higher frequency in our setting and are known to evade relebactam’s inhibitory effect.

At the molecular level, we confirmed that the presence of *bla_KPC* does not always confer resistance to IMR, with 21.1% of KPC-producing Enterobacterales in our study remaining resistant. This suggests that factors beyond the mere presence of the gene – such as high enzyme expression levels or porin alterations – can modulate IMR efficacy (Haidar et al., 2017). Indeed, recent findings have shown that overproduction of KPC-2 can significantly elevate imipenem MICs, reducing the protection offered by a fixed concentration of relebactam (Livermore et al., 2020). In contrast, isolates harboring bla_OXA-48 exhibited a markedly higher resistance rate to IMR (88.9%), consistent with previous evidence that relebactam is ineffective against class D carbapenemases. Avibactam (in the ceftazidime-avibactam combination) can inhibit OXA-48-like enzymes, but relebactam (like vaborbactam) lacks activity against this class. Thus, OXA-48 producers in our cohort behaved essentially as imipenem-resistant, explaining the poor IMR susceptibility in those cases. These nuances illustrate the importance of characterizing not only which β-lactamase genes are present but also their expression and class, as they directly impact inhibitor-based therapies.

Of note, meropenem–vaborbactam (MEV) is an important comparator in KPC-predominant settings. In a recent U.S. surveillance of multidrug-resistant Enterobacterales, MEV retained very high in-vitro activity (≥99% susceptible overall), with KPC the dominant carbapenemase among CRE isolates (Shortridge et al., 2023). Clinically, the randomized TANGO II trial reported higher clinical cure and lower mortality with MEV versus best available therapy for CRE infections (Wunderink et al., 2018). Mechanistically, vaborbactam is a cyclic boronate inhibitor with potent activity against class A (including KPC) and many class C β-lactamases, but it does not inhibit class B metallo-β-lactamases or class D OXA-48-like enzymes—hence activity depends on local mechanism epidemiology. Consistent with current guidance, MEV is favored for serious infections due to KPC-producing Enterobacterales, whereas it is not expected to cover MBL- or OXA-48-like producers and has variable utility for difficult-to-treat *P. aeruginosa*. Given our cohort’s sizeable proportion of MBL and OXA-48-like producers, the expected benefit of MEV in our setting is likely lower than in KPC-predominant regions, reinforcing the need for mechanism-directed therapy.

For *P. aeruginosa*, our data suggest a more complex resistance phenotype. Despite a low frequency of acquired carbapenemase genes, 74% of isolates were resistant to IMR, indicating that alternative mechanisms—such as porin loss and efflux pump overexpression—predominate in this species (Livermore et al., 2020). This highlights the limitations of relying solely on carbapenemase gene detection when evaluating treatment options for *P. aeruginosa*. Recent genomic analyses of IMR-resistant *P. aeruginosa* high-risk clones support these observations. In a study from China, nearly all IMR-resistant ST463 *P. aeruginosa* isolates were found to produce KPC-2 (with significantly elevated *bla_KPC-2* copy number and expression), while IMR-resistant ST235 isolates predominantly harbored the inhibitor-insensitive *bla_GES-5* carbapenemase (Wang et al., 2023). Notably, cloning experiments showed that imipenem resistance conferred by GES-5 was largely unaffected by relebactam, underscoring how certain non-KPC enzymes can circumvent IMR therapy (Wang et al., 2023). Apart from acquired β-lactamases, *P. aeruginosa*’s intrinsic resistance mechanisms are critical. Carbapenem resistance in this organism frequently arises from derepression of its chromosomal AmpC (PDC) together with loss of the OprD porin (especially impacting imipenem), or from upregulation of efflux pumps (e.g., *MexAB-OprM*) often in concert with porin loss (Livermore et al., 2020). Such mechanisms likely explain the high IMR resistance in our *P. aeruginosa* isolates that lacked carbapenemase genes. Therefore, comprehensive diagnostics for *P. aeruginosa* should include phenotypic assessments (e.g., IMR susceptibility tests) in addition to molecular tests for carbapenemases, to avoid false security when a gene panel is negative. Our findings reinforce that a multifaceted approach is needed to predict *P. aeruginosa* resistance: molecular surveillance should be coupled with tests that can detect porin deficiencies or efflux-related resistance (for instance, carbapenem–β-lactamase inhibitor synergy tests or advanced rapid diagnostics) to guide therapy effectively.

The NGS analysis further corroborated the multidrug-resistant nature of circulating *K. pneumoniae* clones, revealing concurrent resistance to aminoglycosides, colistin, and fluoroquinolones (Livermore et al., 2020). These isolates also harbored several virulence-associated genes, potentially contributing to their successful dissemination and pathogenicity. The sequenced isolates showed a complex resistome dominated by carbapenemase genes (e.g., *bla_KPC-2, bla_KPC-3, bla_OXA-2*) and extended-spectrum β-lactamases (*bla_SHV-11, bla_TEM-1, bla_CTX-M-14*), conferring broad resistance to β-lactams. Additional resistance genes explained resistance to aminoglycosides [*aac(6′)-Ib, rmtB1*], colistin (*pmrB_R256G, mgrB_C28Y*), fluoroquinolones (*gyrA_S83I, parC_S80I*), TMP-SMX (*dfrA, sul1/2*), macrolides [*mph(A), erm(B)*], and other antibiotics like fosfomycin (*fosA5*) and chloramphenicol (*catA1, floR2*). Virulence profiling revealed the presence of genes related to iron acquisition (*ybtP, ybtQ, iutA*), capsule production (*magA, rmpA*), fimbrial adhesins (*fimH, mrkD*), and serum resistance (*traT*), supporting enhanced biofilm formation, immune evasion, and systemic dissemination. These findings underscore the therapeutic challenge posed by such multidrug-resistant (MDR) *K. pneumoniae*, especially in the context of co-resistance to last-resort agents and augmented virulence. The convergence of resistance and virulence factors observed here is particularly alarming, as it echoes recent reports of carbapenem-resistant hypervirulent *K. pneumoniae* emerging in various regions (Kanj et al., 2022; Tängdén and Giske, 2015). These hypervirulent MDR clones have caused hospital outbreaks with high mortality, presenting unprecedented challenges for infection control. Our data highlight that Brazilian hospitals are not exempt from this threat – the high prevalence of virulence factors in our isolates suggests a potential for severe, invasive infections and rapid spread. This reinforces the importance of rigorous infection control measures (e.g., contact precautions, cohorting, and environmental decontamination) alongside antimicrobial stewardship to prevent dissemination of these dangerous clones (Tängdén and Giske, 2015).

Our study has evident limitations. This multicenter cohort is region-specific in Brazil, and enriched for carbapenem-resistant isolates, which may overestimate resistance compared with unselected populations. We did not directly phenotype porin loss or efflux activity, which likely contribute to IMR resistance in *P. aeruginosa*. Some species had small sample sizes, limiting precision. Finally, our PCR panel, although expanded, may miss rare or emerging carbapenemases.

In summary, our study confirms the significant burden of carbapenem-resistant Enterobacterales and *P. aeruginosa* in Brazil and underscores the challenges posed by emerging resistance to IMR. These findings support the need for routine molecular surveillance, rational antibiotic stewardship, and the development of novel therapeutic strategies to overcome resistance mechanisms that compromise IMR (Kanj et al., 2022; Tängdén and Giske, 2015). Notably, pathogens harboring class B or D carbapenemases – which are largely impervious to relebactam – will require alternative treatment approaches. In the case of KPC- and OXA-48-producing Enterobacterales, ceftazidime-avibactam has become an important option, although resistance via KPC variants is an evolving concern. For MBL-producing strains, two recent additions to the arsenal stand out: cefiderocol, a siderophore cephalosporin with potent activity against many MBL-producers, and the combination of aztreonam with avibactam, which together can neutralize metallo-β-lactamases and serine β-lactamases and is now recommended by international guidelines for such difficult pathogens (Kanj et al., 2022). Furthermore, emerging β-lactam/β-lactamase inhibitor combinations are in late-stage development, aiming to fill the gaps in our armamentarium. These include agents combining a β-lactam with novel boronate inhibitors like taniborbactam (active against classes A, C, D, and some B enzymes) and others pairing β-lactams with next-generation DBO inhibitors like zidebactam or nacubactam (Kanj et al., 2022). Early studies indicate these agents may extend coverage to organisms that currently evade IMR therapy. As these new therapies become available, rapid and precise diagnostics will be crucial to identify the resistance mechanism present in each isolate and to tailor treatment accordingly. For example, lab tests that quickly distinguish KPC producers from MBL producers (via PCR or novel phenotypic assays) can direct clinicians to use IMR or instead an MBL-active regimen (such as aztreonam-based combinations) without delay. Finally, our findings emphasize that aggressive infection control remains essential (Tängdén and Giske, 2015). Preventing the spread of IMR-resistant bacteria – especially those that are pan-resistant or carry enhanced virulence – requires a multifaceted approach including screening of high-risk patients, strict adherence to isolation protocols, and outbreak preparedness. By combining advanced therapeutic options with vigilant surveillance and infection control, healthcare systems can better mitigate the threat posed by IMR-resistant Enterobacterales and *P. aeruginosa* in Brazil and beyond.

In conclusion, our multicenter analysis of carbapenem-resistant isolates from Brazilian hospitals shows that IMR activity is substantially constrained in this setting: about one-third of *K. pneumoniae* and nearly three-quarters of *P. aeruginosa* were IMR-resistant. Among Enterobacterales, KPC remained predominant, but NDM increased, and IMR resistance was especially frequent in OXA-48–like and MBL producers; notably, a meaningful subset of KPC producers also resisted IMR, suggesting additional permeability/expression mechanisms. In *P. aeruginosa*, high IMR resistance occurred largely in the absence of acquired carbapenemases, consistent with porin loss and efflux. Overall, these findings argue for routine molecular surveillance and mechanism-directed therapy (rather than agent-directed empiricism) to optimize outcomes and stewardship where MBLs and OXA-48–like enzymes are emerging.

## Data Availability

The raw data supporting the conclusions of this article will be made available by the authors, without undue reservation.
